# Tuberculosis incidence in Portugal: spatiotemporal clustering

**DOI:** 10.1186/1476-072X-6-30

**Published:** 2007-07-11

**Authors:** Carla Nunes

**Affiliations:** 1 Epidemiology and Statistics Group, National School of Public Health, Avenida Padre Cruz, 1600- 560 Lisboa, Portugal

## Abstract

**Background:**

The statistics of disease clustering is one of the most important tools for epidemiologists to detect and monitor public health disease patterns. Nowadays, tuberculosis (TB) – an infectious disease caused by the *Mycobacterium tuberculosis *– presents different (development in populations and antibiotics resistance) patterns and specialists are very concerned with it and its association to several other diseases and factors. Each year, tuberculosis kills about three million people in the world. In particular, it is responsible for the death of more than one-third of HIV-infected people, who prove particularly susceptible due to a decline in their immune defences. The purpose of this study is to determine if there are spatiotemporal tuberculosis incidence clusters in continental Portugal. The presented case study is based on the notification of new tuberculosis cases (disease incidence), between 2000 and 2004. In methodological terms, the spatial scan statistic, used to identify spatiotemporal clusters, was improved by including two new approaches: definition of window sizes in the cluster scanning processes considering empirical mean spatial semivariograms and an independent and posterior validation of identified clusters (based on geostatistical simulations).

**Results:**

Continental Portugal is organized in 18 districts with 278 sub-districts. For this case study, the number of new notified cases of TB, per sub-district and per year (2000–2004) was available. TB incidence presents clear spatial patterns: a semivariogram consistent with 40% of nugget effect and 60% of spatial contribution, following an exponential model with a range of 143 kilometres. Temporal semivariograms were not conclusive, as only 5 years of data were available. The spatial and temporal persistence of clusters were analyzed considering different models. Significant high incidence rate space-time clusters were identified in three areas of Portugal (between 2000 and 2004) and a purely temporal cluster was identified covering the whole country, during 2002.

**Conclusion:**

In terms of spatiotemporal clustering of tuberculosis disease, the proposed methodology allowed the identification of critical spatiotemporal areas. In Portugal there were 3 critical districts (Porto, Setúbal and Lisbon) with high rates of notified incidences between 2000 and 2004. In methodological terms, semivariogram parameters were successfully applied to define spatiotemporal scan window sizes and shapes (ellipsoidal cylinders), showing very good results and performances in the case study. After defining the clusters, these were authenticated through a validation method, based on geostatistical simulations.

## Background

In 1993, the World Health Organization (WHO) declared tuberculosis (TB) to be a global emergency. Disease control is based on effective diagnosis, treatment and monitoring of TB cases, and must undergo a directly observed short course therapy [1]; this defines "DOTS strategy". Some authors sustain that tuberculosis can be controlled only if appropriate policies are followed and effective clinical and public health management is ensured [2]. New perspectives and ways of addressing TB treatment and control are needed.

Disease mapping has a long history [3] and it is not surprising that this method of descriptive analysis was first used as an attempt to identify sources of infections and to describe rates of spread. The description of spatial/temporal patterns of disease incidence and mortality can be defined as geographical epidemiology. This can be considered as a part of descriptive epidemiology, which is more concerned with describing the occurrence of diseases with respect to demographic characteristics (*e.g*. age, race and sex), place and time [4]. In the past decade, several studies on geographical epidemiology have been published all over the world. Porter [5] published a very interesting study about Geographical Information Systems (GIS) and the tuberculosis DOTS strategy. Moonan *et al*. [6] used GIS technology to identify areas of tuberculosis transmission and incidence in the USA, 1993–2000. Rodrigues-Jr *et al*. [7] studied spatial distribution of M. tuberculosis-HIV co-infection in São Paulo State, Brazil, 1991–2001. In India, Tiwari *et al*. [8] investigated geo-spatial hotspots for the occurrence of tuberculosis in the Almora district, using GIS and spatial scan statistics. In the WHO European region, the TB situation is critical in 16 of the 51 countries, with a resurgence of the disease and a dramatic increase in notification rates during the last 10 years [9]. Serra *et al*. [10] studied the tuberculosis surveillance and evaluation system in Portugal. Antunes *et al*. [11] took a historical perspective on the 1994 tuberculosis situation in Portugal. Briz [12] studied the country's effectiveness of the Tuberculosis Control Program, stressing that, while Portugal has the highest notified incidence of tuberculosis in Western Europe, the estimated case detection is one of the best (which may artificially contribute to the relatively bad incidence score). The General Directorate of Health publishes an annual monitoring report about incidence and prevalence of TB in Portugal, based on these epidemiologic concepts and using GIS only to allow visual geographical interpretations.

In my opinion, the identification of geographical areas with on-going disease transmission, using GIS and spatiotemporal statistical analyses, has become indispensable. Spatiotemporal clustering methods are concerned with the identification of greater density of occurrences of a phenomenon in certain places at certain times. These techniques have been intensively applied in several areas such as demography, criminology, toxicology, among others.

Disease clustering is a technique of major interest to epidemiologists which has been studied for many decades; for an effective disease management it is essential to know when, where and to what degree a disease is present. During the last decade there has been a huge and fast development of spatiotemporal clustering applied to health: assessment of infectious diseases, cancer, rheumatisms, diabetes and accidents, among others.

In 1995, Kulldorff and Negarwalla [13] developed a new method for the detection and inference of spatial clusters for a particular disease, with a clearly defined hypothesis test and test statistics based on the likelihood ratio. Klassen *et al*. [14] studied geographic clustering of prostate cancer. Nkhoma *et al*. [15] detected spatiotemporal clusters of accidental poisoning mortality. Recently, Kadafar *et al*. [16] presented a compilation of several methodologies to detect disease clusters in time and/or space. Sheridan *et al*. [17] investigated the distribution of bovine spongiform encephalopathy (BSE) in herds of cattle in Ireland between 1996 and 2000, prior to the introduction of widespread active surveillance. Due to the exponential growing and development of this subject, review papers of several methods of spatial and (more recently) spatiotemporal clustering, have been particularly welcome [18-23].

Conjugation between classical approaches of space-time clusters and geostatistical methodologies are relatively recent. Berke [24] used kriging to estimate spatial risk functions from regional count data. Goovaerts and Jacquez [25] present an application of spatially correlated neutral models (based on Sequential Gaussian Simulation) for the detection of changes in mortality rates across space and time using the local Moran I Statistics. Goovaerts [26] used Poisson kriging and p-field simulation to assess cancer mortality risk.

In the present study, the objective is to map spatiotemporal tuberculosis incidence in Portugal in order to determine when and where unusually high concentrations of new cases occurred, considering the gender distributions in local populations. Spatiotemporal clustering adjusted with geostatistical parameters is used.

Two important perspectives were considered in this case-study, as cluster detection was done both retrospectively, trying to define when and where the clusters could be found, and prospectively, given that the data set is generated as an on-going process, with data for recent cases added as the cases are reported. With each inclusion of new data, the prospective analysis addresses the question if there is a new cluster emerging.

## Methods

Different approaches for the detection of space-time clusters have been proposed and implemented. The most referred approach relies on the space-time scan statistic [27], which identifies the most significant cluster of a particular shape in space and time. This method identifies the zone showing the strongest evidence of representing a high density cluster. The scan statistic is based on a maximum likelihood ratio for each potential cluster that expresses how much more likely the observed density is, under the hypothesis of clustering, than under the hypothesis of uniformity. Since the exact distribution of the test statistic cannot be determined, Monte Carlo simulation is used to perform the hypothesis test.

One important aspect of this cluster detection method is the choice of cluster shapes and cluster dimensions. This choice will obviously directly influence the final results. Kulldorff [27] referred that the best choice of window depends on the application and indicated some possibilities: all circular, with all circles centred at any of several foci on a fixed grid, with a possible upper limit on circle size or with a fixed circle size; all rectangles of a fixed size and shape; and, when looking for space-time clusters, the possibility of using cylinders, scanning circular geographical areas over variable time intervals.

Iyengar [28] analysed the influence of cluster shape and concluded that cylindrical or elliptical search windows can limit the fit of models, proposing analyses with more than one shape, computing, for instance, square pyramidal shapes.

The first problem addressed here is not cluster shapes (circular and elliptical shapes are considered), but cluster dimensions. Using only cylindrical shapes, a large number of windows (with different radii), can be computed. If we consider the hypothesis of elliptical windows, the number of possible windows to test grows fast (each window is defined by centre coordinates, angle (azimuth), major axis dimension and minor axis dimension). To deal with this infinity of possibilities, all software must have some parameters defined by default. For instance, when elliptical spatial scan statistics are requested, SaTScan, (one of the most popular and free space-time clustering software, [29]) uses the circular window plus four different elliptical shapes (by default).

The idea of this paper is that disease-specific semivariogram parameters can be used to infer these windows parameters. In simple terms, a semivariogram can be defined as a key function in geostatistics that describes spatial and/or temporal patterns of the observed phenomenon. It has a long and exhaustive history in scientific geostatistical studies [30-32].

The semivariogram represents an average behaviour continuity (mean pattern) for the whole study area, whereas a cluster is determined by the behaviour in a specific place (local pattern). But, although there is no evidence about any relation of global mean patterns with local behaviours, the use of mean pattern information is certainly more sensible than using some default case-study independent parameters. For instance, the software SaTScan [29] is parameterized by default (in advanced options including elliptical windows) to scan circular windows plus four different elliptical shapes, with ratios of the longest to the shortest axis of the ellipse of 1.5, 2, 3, 4 and 5. For each shape, different numbers of angles of the ellipse are tested: 4, 6, 9, 12 and 15, respectively. The north-south axis is always one of the angles included, and the remaining angles are equally spaced around the circle. For each shape and angle, all possible sizes of the ellipses are used, up to an upper limit specified by the user in the same way as for the circular window. But the question here is: Why these defaults? They don't have any parameterization related to the specific case-study.

Semivariograms describing the mean space-time patterns can be useful in this context, even when, like in this case-study, it is only possible to compute mean spatial semivariograms [33,34]

Assuming a value *Z*(*x*_*i*_, *t*_*j*_) of variable *Z*, observed in a certain sub-district i (represented through a geometric central point *x*_*i*_) for time *t*_*j*_, this value can be correlated with the incidences observed in previous time periods for the same area, and with incidents observed at neighbouring sub-districts during the same or previous time periods.

The spatial continuity for a given period of time can be characterized using a mean spatial semivariogram, *γ*_*s*_(*h*), computed by averaging the spatial semivariogram of each time *t *slice and representing the mean spatial pattern for that given period of time [34]:

γs(h)=12NtNh∑j=1Nt∑i=1Nh[Z(xi,tj)−Z(xi+h,tj)]2
 MathType@MTEF@5@5@+=feaafiart1ev1aaatCvAUfeBSjuyZL2yd9gzLbvyNv2CaerbwvMCKfMBHbqedmvETj2BSbqee0evGueE0jxyaibaieIgFLIOYR2NHOxjYhrPYhrPYpI8F4rqqrFfpeea0xe9Lq=Jc9vqaqpepm0xbbG8FasPYRqj0=yi0lXdbba9pGe9qqFf0dXdHuk9fr=xfr=xfrpiWZqaaeaabiGaaiaacaqabeaabeqacmaaaOqaaGGaciab=n7aNnaaBaaaleaacaWGZbaabeaakiaacIcacaWGObGaaiykaiabg2da9maalaaabaGaaGymaaqaaiaaikdacaWGobGaamiDaiaad6eacaWGObaaamaaqahabaWaaabCaeaadaWadaqaaiaadQfadaqadaqaaiaadIhadaWgaaWcbaGaamyAaaqabaGccaGGSaGaamiDamaaBaaaleaacaWGQbaabeaaaOGaayjkaiaawMcaaiabgkHiTiaadQfadaqadaqaaiaadIhadaWgaaWcbaGaamyAaiabgUcaRiaadIgaaeqaaOGaaiilaiaadshadaWgaaWcbaGaamOAaaqabaaakiaawIcacaGLPaaaaiaawUfacaGLDbaadaahaaWcbeqaaiaaikdaaaaabaGaamyAaiabg2da9iaaigdaaeaacaWGobGaamiAaaqdcqGHris5aaWcbaGaamOAaiabg2da9iaaigdaaeaacaWGobGaamiDaaqdcqGHris5aaaa@6261@

where *Nt *is the number of time periods and *Nh *the number of pairs of sub-districts at distance *h *from each other (geometric central points distances).

These are commonly represented as a graph that shows the variance behaviour *γ*(*h*) against the (distance or time) lag *h*. Usually and in presence of spatial/temporal dependencies, the semivariogram initially rises from some point on the *y *axis (nugget effect) and reaches a threshold (sill) at a certain location (defining the range).

Ranges of the adjusted semivariogram models as well as angles (azimuths), can provide a first approach to case-study specific scan window parameters. Here, the use of the semivariogram parameters is proposed to infer a possible window shape, following the assumption (or expectation) that local behaviours can influence or be represented by the global behaviour's parameters, (not following their exact values, but their general shape – angle and ratio of the longest to the shortest axis of the ellipse). So, in order to incorporate case-study coherence into the parameters definition, these parameterized elliptic windows were used next to circular windows. This way, the number of different scan windows tested can be reduced and will follow a case-study specific parameterization.

The second problem addressed is related with the validation of identified clusters. Goovaerts and Jacquez [25] and Goovaerts [26] have presented some developments in neutral models, stressing that spatially uncorrelated models can lead to some predisposition to reject the null hypothesis, defining false clusters. Here a different approach is proposed: After cluster identification, (using hypotheses based on spatially uncorrelated models – Monte Carlo) and knowing about the existence of a specific spatial/temporal pattern, geostatistical simulations – Sequential Gaussian Simulations (SGS, [25,33]) – were computed. SGS is used to generate realizations for each identified cluster, not considering the incidence rates observed in this cluster and imposing the fitted semivariogram model. "True" clusters (identified using spatially uncorrelated models) must present extreme (high) observed rates in their simulated local distributions (conditioned to the semivariogram and to neighbouring incidence rates).

For each cluster, the validation process can be summarized in the following steps:

- Temporally, delete observed incidence values of all spatiotemporal points within the cluster (sub-districts/years);

- Simulate *k *scenarios for all these points, using SGS;

- For each scenario, sum up simulated spatiotemporal values for the same spatiotemporal observed location (within this cluster);

- Compute a local distribution with *k *global incidence values;

- Compare the global observed incidence value (sum of observed incidence values within this cluster) with the local distribution: compute the probability of the simulated notified rates being above the observed notified rates of this cluster.

This validation process (considering only the spatiotemporal continuity after cluster identification) is a different and simpler approach to deal with the potential tendency to reject the null hypothesis defining false clusters. Note that this processes requires the reproduction of the mean spatial semivariogram inferred from all data, but only the histogram of those data considered in the simulation process (without data belonging to cluster). For each cluster, each simulated scenario is computed in a space-time domain (considering time as a third spatial axis).

## Results

### Case study

Portugal is fully covered by the National Tuberculosis Control Program (PNT), which strictly follows the World Health Organization's Strategy [1] defined as Directly Observed Therapy, Short Course (DOTS) and regularly issues a progress report. The information used, regarding cases notified between 2000 and 2004, was provided by SVIG-TB, the specific PNT information system managed by the PNT coordination team, at the general Directorate of Health. Data are all related to incidence notifications. Population denominators come from official statistics, issued by the National Statistics Institute. This study is not a population-based study, but an institution-based study, as it relies on the compulsory notification of cases to the General Directorate of Health, by clinicians According to Briz [12], Portugal presents a detection rate of 83% (regarding pulmonary cases).

By analysing reported incidence rates per sub-district and per year, instead of the number of cases only, spatial variability of population size is accounted for. Continental Portugal is organized in 18 districts with 278 sub-districts (spatial unit). The temporal aggregation is on an annual basis (5 years: between 2000 and 2004).

**Figure 1:**
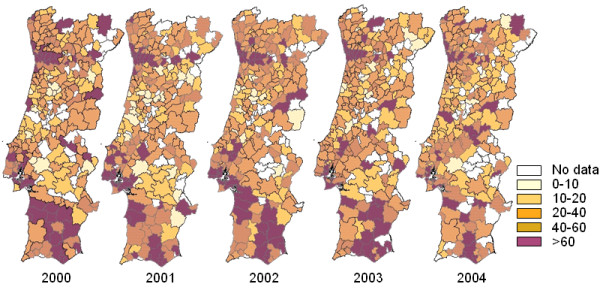
Notification rates (/10^5^) per sub-district and per year.

Figure 1 shows the notified incidence rate per year and per sub-district.

At a sub-national level, assuming a homogeneous detection rate, geographic heterogeneity of notified incidence rates is clearly present.

**Figure 2:**
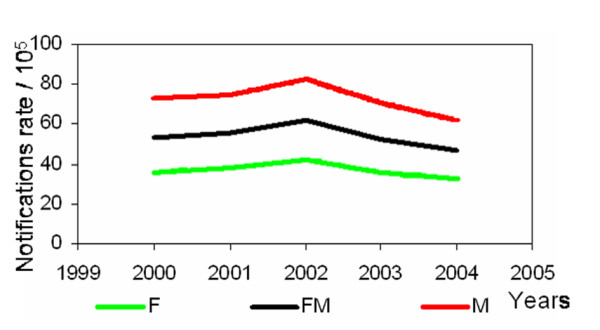
Notification rates (/10^5^) per year, for the whole population (FM) and by gender.

Figure 2 shows the incidence rate per year for the whole population (FM) and by gender (F = Female, M = Male).

**Figure 3:**
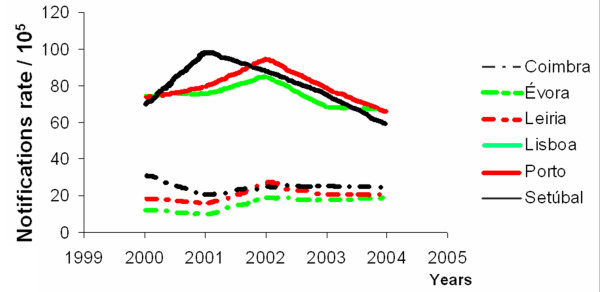
Notification rates (/10^5^) per year, for select districts.

In this first approach, space-time clustering is based on the notified cases per sub-district per year. Figure 3 presents the yearly incidence rates for the three Portuguese districts with the lowest and the three with the highest incidence rates.

In the districts with the lowest incidence rates, the temporal patterns seem stable. The other districts present decreasing behaviours from 2002 onwards (except for Setúbal), agreeing with the 1972–2002 temporal patterns [12].

**Figure 4:**
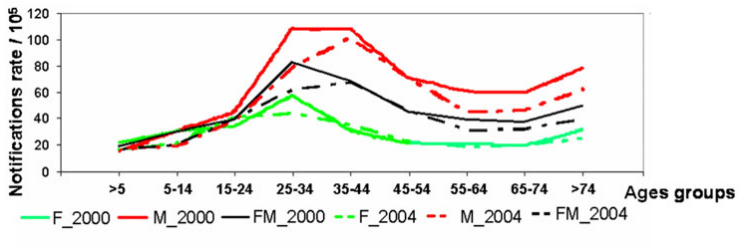
Notification rates (/10^5^) per age group and gender, for 2000 and 2004.

Figure 4 presents the age/gender distribution in the notified incidence data, for the years 2000 and 2004 (as examples).

**Figure 5:**
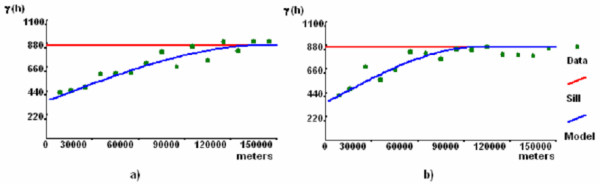
**Mean spatial semivariograms of most representative directions**. Semivariogram a) corresponds to the 90 degrees semivariogram (major range) and semivariogram b) corresponds to the perpendicular 0 degrees semivariogram.

On their own scale, male and female population display similar patterns for the same year. Between 2000 and 2004 a shift of the mode to the next age-group (25–34 to 35–44) can be observed. A tentative explanation, could be that in 2004 the notified cases were more related to reactivations (older ages) and less to new cases, than in 2000. If we interpret this as a temporal tendency, it could point at a successful control program, although this inference is not possible based on a data series of 5 years only. To define directions with major continuity patterns, mean spatial semivariograms were computed for several directions (0, 10, 20, 30, 40, 50, 70 and 80) and also for their perpendicular angles. Figure 5 shows the semivariograms for the most representative directions.

According to the experimental mean spatial semivariograms presented in Figure 5, notified incidence rates have clear spatial patterns. In this case-study, the fitted model has a high nugget effect (350/865 = 40% of variance behaviour, defining a considerable spatially uncorrelated variation or some noise effects) and a spherical structure (responsible for 515/865 = 60% of variance behaviour). Defining the ellipsoids' characteristics, this model shows a geometric anisotropy (the dispersion patterns are not equal in all directions), with a major axis or range of 143 km (maximum distance of spatial correlation), over the 90 degrees azimuth and with an anisotropic ratio of 1.6 (ratio between major axis and minor axis). As already referred, with 5 years of data (on an annual basis) it is not possible to characterize temporal patterns.

### Cluster Identification

In epidemiologic studies it is usually interesting to identify disease clusters, after having adjusted for spatial variations in the density and the characteristics of the background population itself.

In the present study spatiotemporal clustering was performed using the Poisson model, with and without adjustments for a gender covariate (using indirect standardization [35,36]). Due to incomplete information (population denominators per age, per sub-district), the corresponding analysis using age distributions were not possible.

Regarding the shape and dimension of the scan windows, two different approaches were taken: using elliptical scan window defaults (circular and elliptic space-time scan statistic) and using windows depending on semivariogram parameters (and also considering circular shapes). In a spatiotemporal context, this means that circular cylinders and ellipsoidal cylinders were used.

Considering the presence or absence of the gender covariate and default scan window parameters (SaTScan defaults for the elliptic scan statistics), the first two models were defined: model I – without covariates and using scan window defaults; and model II -with gender covariate and using scan window defaults.

For the second approach, window shape and dimension are conditioned by the semivariogram parameters, searching only ellipses with a ratio of 1.6 between the axes in North-South and East-west direction, defining: model III – without covariates; and model IV – with gender as covariate. Note that the semivariogram for this case study presents a 40% nugget effect, suggesting a strong micro-scale variation in incidence rates (which can be real, produced by erroneous measurements and/or dependent on geometric central point distances) not explained by the proposed model. Remember that these four models have also circular cylinder options (by default). To enable comparison with classical approaches (using only circular and not allowing ellipsoidal scanning windows) a fifth model was also computed.

For each model, 999 realizations were generated, using Monte Carlo Simulations. Analysis includes purely spatial and purely temporal clusters. All these models were constrained to a maximum spatial cluster size of 50% of the population at risk. Also, no geographical overlap was allowed, as criterion for reporting secondary clusters.

**Figure 6:**
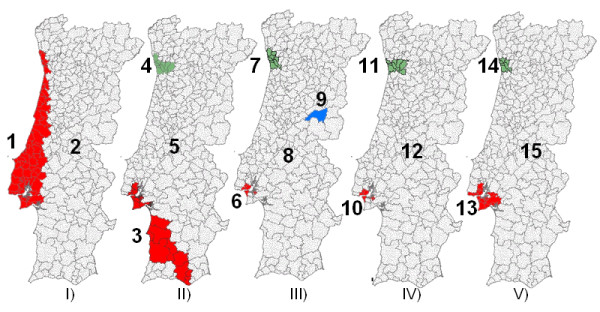
**Mapping the identified spatiotemporal clusters of TB, using the five defined models**. Maps I) and I) were computed using default window parameters, without (Model I) and with (Model II) gender as covariate. III) and IV) were computed using semivariogram parameters, without covariates (Model III); and with gender as covariate (Model IV). Model V uses only circular windows and with gender as covariate.

Detailed characteristics of each cluster (per model) are presented in Table 1. Figure 6 shows the simulation results for the five models. For each model, the most likely cluster is painted with red colour and secondary clusters with green, blue and grey colours.

**Table 1:** Cluster characteristics, for each model.

**Model**	**Cluster**	**Coordinates**	**Minor axis Major axis**	**Angle Ratio**	**Time frame**	***p*-value (LL Ratio.)**	**Obs/Exp**
**I**	**1**	(121667, 292213)	42131.3 210656	84 5	2000–2004	<0.001 (1012.7)	1.27

**II**	**3**	(161776, 109687)	23934.1 118170	-60 5	2000–2004	<0.001 (1033.7)	1.57

**II**	**4**	(154861, 471683)	12881 38643.1	-20 5	2000–2004	<0.001 (697.4)	1.72

**I**	**2**	all	-	-	2002	<0.001 (61.92)	1.14
**II**	**5**						
**III**	**8**						
**IV**	**12**						
**V**	**15**						

**III**	**6**	(110920, 197626)	13024 20838.4	0 1.6	2000–2004	<0.001 (936.9)	1.75

**IV**	**10**						

**III**	**7**	(160762, 475195)	13638.7 21821.9	90 1.6	2000–2004	<0.001 (741.4)	1.68

**III**	**9**	(255455, 350524)	0 0	0 1	2003–2004	<0.001 (22.4)	6.10

**IV**	**11**	(170616, 460108)	15196.8 24314.8	0 1.6	2000–2004	<0.001 (734.7)	1.69

**V**	**13**	(115030, 182346)	28758.64 28758.64	--	2000–2004	<0.001 (954.4)	1.54

**V**	**14**	(159069, 465960)	12945.24 12945.24	--	2000–2004	<0.001 (712.94)	1.71

Comparing the clusters defined by models I and II (Figure 6 and Table 1), these always seem to constitute structures with a ratio of 5 (biggest ratio scanned), generating large and thin ellipsoids. Given that the models only differ in the use of the covariate gender, its influence appears to be significant (different cluster dimensions and identified sub-districts).

A purely temporal cluster was identified by all models: 2002 had high notification rates in the whole country.

Comparing the results obtained with the models III and IV, based on semivariogram parameters (considering or not the gender covariate), we see that the most important areas (including Porto, Lisbon and Setúbal) are identified by both models. But, due to the imposed ratio and angle (1.6 and 90°) the clusters could not extend into other areas. Given that these models differ only in the use of the covariate gender, its influence seems here not significant. Note also that all spatiotemporal clusters have a temporal frame of 2000–2004, including older and not resolved clusters. One cluster composed by only one sub-district (Fundão) and only estimated by model III, was identified for the 2003–2004 period.

Model V, using only circular windows (cylinder shape), defines areas similar to those of models II and IV, but with different dimensions. Note that in elliptical searches, circular windows were also tried but due to the imposed criterion of no geographical overlap (to define only the most significant cluster for each area), circular clusters could not be identified. Clusters 13 and 14 were therefore not identified by model IV, since they were not allowed to overlap geographically with the more significant clusters 10 and 11.

Models III and IV define more specific clusters than the other models, which should be easier to study and to apply prevention actions. It is possible to apply local best practices with lower costs and make more detailed evaluations, if the most critical sub-districts are identified.

In a brief, comparative CPU time analysis: model I takes 11 minutes and 39 seconds to run, model II 12 minutes and 21 seconds, model III runs in 53 seconds and model IV in only 49 seconds. Model V, the most restricted one, runs in 23 seconds. Obviously, these CPU times are directly related with the number of different shapes scanned.

Concisely, the clusters identified here are not in disagreement with the explanatory conclusions drawn from Figures 2 and 3 (Methods Section). There were other identified clusters, all with associated *p*-values greater than 0.9, which are not presented in the results. No significance level was imposed. The presence of extreme *p*-values only in this case-study (lower than 0.001 or greater than 0.9) shows clearly that statistically significant clusters are identified.

### Cluster Validations

To validate the identified clusters, local distributions of the incidence rates were calculated by stochastic simulations (SGS, [25,33]), for each cluster (without taking into account the data of the respective cluster). The idea is to check whether the cluster's data belong to these local distributions. Results are presented in Table 2.

**Table 2:** Cluster validations

**Model**	**Cluster**	**Notification Rate Incidence (Obs.)**	**Probability**
**I**	**1**	14645.65	0.08

**II**	**3**	7912.13	0.08

**II**	**4**	4003.71	<0.001

**I**	**2**	11262.43	0.45
**II**	**5**		
**III**	**8**		
**IV**	**12**		
**V**	**15**		

**III**	**6**	2780	<0.001
**IV**	**10**		

**III**	**7**	5024	<0.001

**III**	**9**	667.99	<0.001

**IV**	**11**	3667.28	0.08

**V**	**13**	5286.38	<0.001

**V**	**14**	2686.355	<0.001

For each cluster, the probability of the simulated notified incidence rates being above the observed notified incidence rates is presented in the last column (Probability). Considering the method and validation process applied, the validation of a purely temporal cluster (2002) cannot be robust: there are no neighbour conditioning points in space to condition local distribution.

Bigger major axis clusters have less high probabilities of presenting extreme values (0.08) than smaller ones. On the other hand, small ellipses tend to validate the identified clusters.

## Discussion

One major restriction of this study, very important and already referred, is that this study is not a population-based study but an intitution-based study; it relies on compulsory tuberculosis incidence declarations to the General Directorate of Health (National Tuberculosis Control Program).

As referred, Portugal presents an estimated detection rate of 83%, but there are no studies about regional heterogeneities of detection rates. These restrictions must be taken into account, as they could produce some biases in the conclusions.

In recent years, the decline of notified TB incidence rates is very slow and not compliant with the goals of a good control program [2]. Notification rate values are twice as large for males than for females. A possible explanation can be explained considering that tuberculosis contamination is directly and strongly related to HIV and risk behaviours, like, alcohol and drug abuse, homelessness, among others, which are more frequent in the male population.

In terms of spatiotemporal clustering of tuberculosis disease the presented methodology allows the identification of critical and very specific spatiotemporal areas. In Portugal there are 3 critical districts (Porto, Setúbal and Lisbon) that present high rates of notified incidence between 2000 and 2004. In comparison to models I and II, models III and IV produce small clusters, which, in epidemiological terms, may be more realistic and useful for prevention and for the application of control policies.

Based on model III (using semivariogram information and no gender information), Fundão, one isolated sub-district was identified as a critical area with high notified tuberculosis rates in 2003 and 2004. This area could benefit from a more detailed evaluation to determine if it represents a real tuberculosis incidence cluster or if the cluster is caused by other factors, for instance, a better notification rate. But, when a variable is found that explains the observed difference, usually no further investigation is well succeeded. This point stresses the importance of performing this kind of studies with adjustments for covariates like age or others, next to gender. In the public health context, the lack of such auxiliary information could constitute a severe restriction of this study.

Nowadays, based on a unreal/isolated concept, tuberculosis could be considered a disease "under control". However, strong relations between the occurrence of this disease and some other diseases or risk factors (HIV, alcohol and drug abuse, homelessness, a.o.) are changing the scenarios, leading to a real increase of incidence rates. These factors must be incorporated in future analysis. We need to discern whether increased incidence rates are the result of improved notification or due to more occurrences.

In methodological terms, the use of semivariogram parameters in the definition of scan window sizes and shapes was successfully applied to this case study and showed very good results and performances. There are several problems associated to models I and II, based on case-study independent random defaults: Are the tested ratios adequate to estimate TB dispersion and clusters? What maximum ratio should be tested? When and how should the parameterization process stop? Do skeletal (big-ratio) shapes make any sense for disease dispersion phenomena? Should we use only circular windows? (Note that, although this option was always available, no circular cluster was identified.) Out of curiosity I tried ratios of 6 and 10, with 12 and 15 angles (sections), respectively, and using the gender covariate. For the ratio of 6 no cluster was identify. For the ratio 10 one cluster was identified (*p*-value < 0.001), which was similar to cluster number 4 in Figure 1 (identified without the gender covariate), though narrower, beginning in the same area (in the south) and continuing northward, along the coastline, until Spain. This is a very confusing result, which proofs the importance and serious consequences of the adopted scan window shapes. Could we accept that the parameterization of the scan window shapes has more influence (or just as much) on disease clustering as the use of covariates?

It is very uncommon to find scientific studies based on elliptical scannings (most use only circular shapes). Comparing the results obtained for models II, IV and V (considering gender), it seems that model IV presents best results to support public health oriented policies.

The clusters presented in this paper were identified based on spatiotemporal independence as the null hypothesis, i.e. assuming that the spatiotemporal distribution of tuberculosis rates is random (without autocorrelation). Considering the semivariogram patterns observed, this assumption does not hold and its application could have influenced cluster identification. However, the fact that, in this case study, *p*-values are very distinct and extreme (below 0.001 or above 0.9) suggests that this did not happen here. Furthermore, posterior and independent validations were conducted, with estimation of local distributions (based on geostatistical simulations) that confirm the identified clusters.

## Competing interests

The author(s) declare that they have no competing interests.
